# Dynamic New World: Refining Our View of Protein Structure, Function and Evolution

**DOI:** 10.3390/proteomes2010128

**Published:** 2014-03-07

**Authors:** Ranjan V. Mannige

**Affiliations:** Molecular Foundry, Lawrence Berkeley National Laboratory, 1 Cyclotron Road, Berkeley, CA 94720, USA; E-Mail: rvmannige@lbl.gov

**Keywords:** protein dynamism, intrinsically disordered, structure, function, promiscuity, evolution

## Abstract

Proteins are crucial to the functioning of all lifeforms. Traditional understanding posits that a single protein occupies a single structure (“fold”), which performs a single function. This view is radically challenged with the recognition that high structural dynamism—the capacity to be extra “floppy”—is more prevalent in functional proteins than previously assumed. As reviewed here, this dynamic take on proteins affects our understanding of protein “structure”, function, and evolution, and even gives us a glimpse into protein origination. Specifically, this review will discuss historical developments concerning protein structure, and important new relationships between dynamism and aspects of protein sequence, structure, binding modes, binding promiscuity, evolvability, and origination. Along the way, suggestions will be provided for how key parts of textbook definitions—that so far have excluded membership to intrinsically disordered proteins (IDPs)—could be modified to accommodate our more dynamic understanding of proteins.

## 1. Introduction

Proteins constitute a diverse class of biomolecules [1] that underlie most of life’s functionalities [2,3]. While the last decade has seen remarkable progress in whole proteome studies, another more silent revolution has been occurring in our understanding of how structural dynamism plays an intricate role in defining the structure, function and evolution of individual proteins. Proteome studies indicate that a good percentage of proteins (over 25%) in complex organisms are highly structurally dynamic [4], with many associated with disease states (such as cancers [5,6], Parkinson’s disease [7], and other afflictions [8]). This has elevated the need to understand the role of structural dynamism and disorder in biology. Here I will attempt to outline the relatively new connections (or newly re-established connections [9,10,11,12]) between structural dynamism, evolvability and function.

Aside from discussing some relevant history and definitions, this review will focus on three “new views” of protein science: (1) structural dynamism—the capacity to be extra “floppy”—is more prevalent in bioactive proteins than previously assumed; (2) structural dynamism allows for high functional/binding promiscuity [13,14,15]; and (3) functional/binding promiscuity plays a big role in the evolution of novel function [16,17]. These features of dynamism fundamentally contribute to the emergence of modular and complex life [4,18]. An inevitable outcome of these relationships is the need to reassess the textbook definitions of what constitutes a protein structure and what differentiates a protein from a peptide. Given the broad implications of these developments, I will refer to a number of useful reviews provided by others. However, while each previous review discusses one part of the picture, I have not found a synthesis of all connections in one venue (e.g., excellent focuses on related ideas, such as enzyme promiscuity [13,19,20], intrinsic disorder [21,22,23,24], and sequence evolution of new function [25] exclude topical discussions on each other). This is the reason of this review; I have attempted to compile these pictures in one venue. Given this aerial-view sketch of the field, the work cited is representative of a larger body.

*Early and Enduring Relationships (a Historical Account)*. To set up the scene for the “new” views discussed later, the following two sub-sections will discuss the traditional relationships between sequence, structure and function (binding). The equally important historical relationships between the evolution of sequence and function will be discussed later in Section 4.2 under evolution.

### 1.1. Sequence → Ordered Structure

Discussed here is the development of the rule that a single biological protein sequence conforms to a specific protein fold, which is the cornerstone of structural biology. Even before the first protein conformation was revealed, Linderstrøm-Lang [26] presented an ordered hierarchical description of protein structure. In this representation, the protein chain, possibly on account of its amino acid sequence (“primary structure”), is able to conform to structurally discrete and ordered motifs (“secondary structures”) that were theoretically shown to exist by Pauling *et al*. [27,28,29,30]. These secondary structural elements, according to Linderstrøm-Lang, would then be arranged compactly into the final functional molecule (“tertiary structure”). Indeed, the first crystal structure [31] did describe a “folded” collection of regular structural motifs—secondary structures—albeit strung together by irregular loops. The picture that a protein sequence could specifically describe snugly-fitted folds (*i.e.*, specific conformations) was strengthened by Anfinsen and Haber’s finding [32] that the amino acid sequence of a protein may dictate the exact tertiary structure that it folds into. These results established the importance of the protein fold (or conformation) as the primary currency for protein activity, which is a rule that has remained largely unchallenged for more than half a century.

### 1.2. Ordered Structure → Specific Function (Binding)

**Figure 1:**
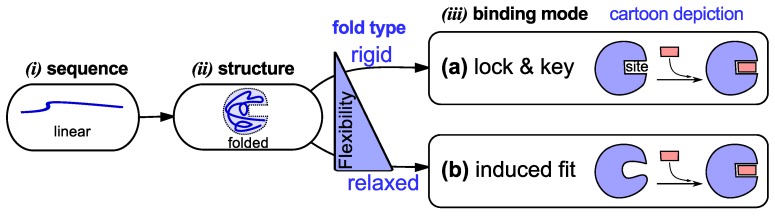
Traditional models of protein structure and function (via binding). In the textbook picture, a linear protein sequence of specific amino acids (***i***) folds into a specific structure or fold (***i***). That fold then binds to its partner by either of two modes (“lock and key” [33,34] or “induced fit” [35]) depending on the fold’s flexibility/rigidity (***iii***).

The view developed in the 1950’s—that specific protein sequences dictate specific folds—suited the then established *lock-and-key* model [33,34] of protein binding (binding is the first and very important prerequisite to protein functionality). The lock-and-key model (Figure 1a) posits that preexisting shape (and, implicitly, chemical) complementarity between a protein and its partner allows for binding to occur [34]. While the lock-and-key mode of protein binding even today matches the working of some proteins [34], Koshland presented an alternative *induced fit* model (Figure 1b) that matched the working of proteins with more pliable binding sites [35]. Induced fit posits that a binding partner is able to induce complementarity within a protein’s binding site, upon which binding occurs [35].

Both models of protein binding (Figure 1) require the existence of a specific fold that either binds to its partner without a fuss (lock and key) or with some convincing (induced fit). This relationship between folded conformation and function (via binding) [2,3] underlies the substantial efforts invested in elucidating conformations via experiment [36] and theory [37]. It is certainly the case that these atomistically-resolved folded conformations have assisted in uncovering a trove of functional and evolutionary understandings [36], while also validating [34,38] the two traditional models of binding. The connection to a folded, structured protein, however, is also the reason that these two models fall short in explaining the binding modes of *dynamic* proteins, discussed next.

## 2. First “New View”: Protein Dynamism and Structure

This section describes how, over decades, exceptions to the neat relationships discussed above—that *specific sequence* dictates *static structure* that dictates *specific function*—have left us with today’s need to reintroduce dynamism as an important factor in protein structure and function.

### 2.1. The Increasingly Disordered View of “Perfect” Protein Structure

The first structure to usher in the age of structural biology—Kendrew *et al*.’s myoglobin [31]—also apprehended well the now traditional view of protein structure: every backbone atom position of the reported myoglobin structure [39] was precise, setting the stage for the one conformation view of protein structure. This “perfect” view of protein structure (where all backbone positions are precisely defined) was supported by the relatively well-formed structures that were reported in the early years of the protein databank (PDB) [36,40]. This is likely because “rigid” proteins are more likely to be crystallized (and then structurally characterized) than floppy ones [41].

Today, however, with the improvement of crystallization and NMR techniques, structures within the PDB display consistent deviation from perfect structure, with ∼5% of amino acids in deposited structures being disordered (invisible) [42]. Computational simulations of folded proteins further painted a more dynamic picture of protein structure [43,44,45].

As if disorder/ambiguity in the solved structures themselves is not enough, a majority of PDB structures were found to cover only small portions of the protein sequence found in nature: as of 2007, ∼40% of PDB structures contain disordered stretches of moderate length (between 10 and 30) [46] and ∼10% display stretches of disorder greater than 30 residues in length [46] (not to mention those proteins that are too dynamic altogether for crystallization [41]); even in the 1980’s, the first spherical virus capsid structure—PDB ID 2TBV—was found to possess a stretch of hundred amino acids missing from the structure [47]. These reports have been priming the field for a more systematic treatment of disorder in *regions* of a protein. Before commencing with these discussions, it is important to first distinguish the idea of a protein structure from protein conformation.

### 2.2. Refreshing the First Textbook Definition: Protein Structure is an Ensemble

While the single-sequence-to-single-conformation picture of proteins gained prominence, other models existed for decades but were not as easily accepted/validated. As early as the 1930s, protein structure has been described as an ensemble of conformations that are inter-convertible and nearly degenerate in energy [11,12,48,49,50]. Despite attempts to describe enzymes as ensembles (reviewed in [10]), theory and experiments from the fifties and early sixties [27,31,32] helped bolster the idea that a protein structure describes a very tight distribution around one particular conformation (or fold), which strengthened the idea that a protein structure *is* a protein conformation (and *vice versa*). The implicit thermal perturbations visible within the protein (as shown by NMR [51]) were considered to be just variations of the average structure. This viewpoint, while suitable for most well-folding proteins, has resulted in substantial resistance to the idea that massive disorder and protein dynamism may play important roles in the protein world.

The discovery, however, of highly dynamic and sometimes even completely disordered proteins (discussed next) requires this single-fold/conformation view of protein structure to be re-addressed. Particularly, the simplification that a protein structure constitutes one fold or conformation must be lifted, and the original description of protein structure—one that never really left the field but took a back seat—must be re-emphasized in structural biology. That is, a structure must only be described as an *ensemble* of conformations (Box 1), folded or not.

BOX 1 **A Conformation Does Not a Structure Become**

**Structure ≠ conformation**
**Structure = ensemble of conformations**
 
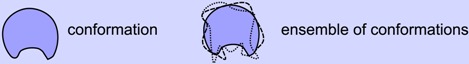
Unlike the synonymous use of the words “structure” and “conformation” in the macroscopic world (e.g., the structure of a building and its conformation/configuration are indistinguishable), at the molecular level, a structure is a *collection* of accessible conformations that together constitute the temperature-dependent native state structural ensemble. The native state ensembles of some proteins are more diverse than others, making some proteins more dynamic than others.

### 2.3. Intrinsically Disordered Regions (IDRs) Are Important to Function

Many dynamic loops that were thought to be byproducts of folded structures (or connectors between secondary structures [26]) are now known to provide crucial molecular functionality (especially promiscuous functionality; Section 3). An example of such a protein is ubiquitin, which, while describing a general fold, displays a *region* that is structurally highly variable that binds specifically to more than fifty distinct proteins [52,53]. The strengthening relationship between disordered regions and function, along with the prevalence of disorder in the PDB [46], have fueled a renewed focus in the role of disorder in structure and function.

### 2.4. Intrinsically Disordered Proteins (IDPs) Bolster the Dynamic View

In addition to folding proteins that display highly dynamic/disordered regions [46], another class of proteins called intrinsically disordered proteins (IDPs) [14,54,55,56,57,58,59,60,61] push the envelope when it comes to expanded structure ensembles. IDPs were discovered only in the last couple decades, in large part due to improved experimental methods for observing intrinsic disorder [54,59,62] and breakthroughs in picking out sequences and regions of sequences that code for intrinsic disorder [4,15,18,63,64,65,66]. Today, IDPs are believed to have little-to-no well-defined conformations in their structural ensemble (*i.e.*, no persistent conformation exists, although residual structure *is* believed to exist and be of indispensable value to binding [67,68]; Section 3). Interestingly, even without a single stable and dominant conformation, these proteins *do* display biologically relevant binding events, where the presence of the right partner often results in the collapse of the various structural possibilities into dominantly one conformation complementary to the partner [24] (Section 3).

*A protein with many names.* While the name “intrinsically disordered protein” or the acronym “IDP” has been gathering popularity and consensus, the last decade witnessed a number of names that were initially given to this class of protein including binary combinations of the two sets of words {natively, inherently, intrinsically, exceptionally, naturally} and {denatured, unfolded, unstructured, disordered, flexible} (reviewed in [69,70]). The exciting introduction of the journal *Intrinsically Disordered Proteins* this year [70] indicates that the field is equilibrating to a common term (intrinsically disordered protein or IDP), and, that the steady confluence of concepts, methods and goals regarding IDPs are maturing well, making the coming decade an exciting one for *un*structural biology [71].

With these dynamic additions to the protein repertoire (Box 1) a number of new modes of protein binding have emerged, which will be discussed soon. But first, from today’s textbook definitions of what makes a protein (versus a peptide), these dynamic proteins are illegitimate. This must change.

### 2.5. Refreshing the Second Textbook Definition: Proteins *versus Peptides*

*What distinguishes proteins from peptides?* Given the established relationship that a protein first folds and then functions, the idea prevailed for decades that proteins are distinguished from peptides primarily because of their capacity to fold [72] and secondarily by their longer lengths [2]. Then came small folded protein domains (such as the zinc finger domain [73,74]) and long dynamic proteins such as those discussed above [24,64]. These rule-breakers to the canonical definition of a protein, while first considered to be exceptions, today constitute a substantial portion of the proteome (e.g., over 25% of eukaryote proteins are intrinsically disordered [4]). These accumulating “exceptions” must compel us to revise the distinction between protein and peptide, as these textbook distinctions are imparted at the undergraduate level onwards and serve as schisms to understanding the true nature of a whole proteome. I therefore propose a new set of definitions for what distinguishes a protein from a peptide (Box 2), partly because no general consensus exists today, as far as I am aware.

BOX 2 **Distinguishing Proteins from Peptides**

Both peptides and proteins are linear chains of amino acids. What distinguishes a protein from a peptide?
 
**Old distinction:** A protein is a **long** (>50 amino acid [2]) peptide chain that **folds** [72] reliably into a single (or few) distinct conformation(s).
 
**Expanded distinction:** A protein is a peptide chain that **folds** [72] reliably into a single (or few) distinct conformation(s) *or* **binds** reliably to at least one specific cognate partner.
 
The new view assigns highly dynamic, often disordered, but still functional chains—a legitimate entity in the proteome—as legitimate within the protein family.

## 3. Second “New View”: Protein Dynamism and Promiscuous Function

**Figure 2:**
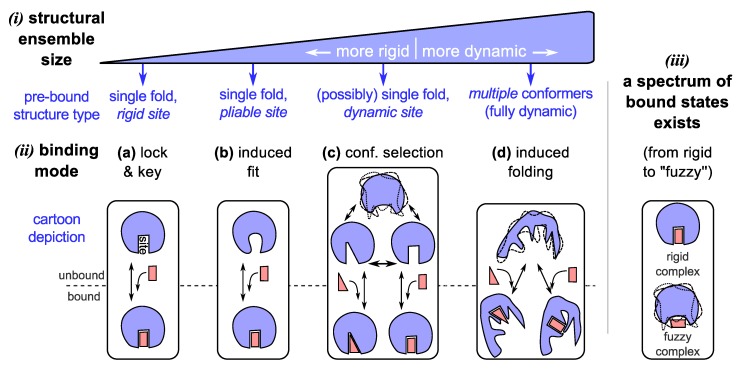
The dynamic “new view” of protein structure and function. In the “new” view, a protein chain occupies an ensemble of conformations as its structure (***i***) by virtue of its amino acid sequence and environment. Depending on the number of conformations and capacity to cycle through the various conformations, we arrive at a spectrum of dynamic proteins that range from relatively rigid (well-folding proteins describing only one major average conformation) to highly dynamic and extended (such as intrinsically disordered proteins). The dynamism of a protein often dictates the modes of binding available to it (***ii***), which is a crucial requirement of most functioning proteins. Given an ensemble view of structure, while traditional binding modes such as lock-and-key (**a**) [75,76,77] and induced-fit (**b**) [78,79,80] are accommodated, “new” modes of binding emerge for dynamic (**c**) [9,81,82] and intrinsically disordered (**d**) [62,83,84,85,86] proteins, which allow for promiscuous functionality. In addition to binding modes, bound configurations display a range of dynamic states (***iii***) [87,88], which provides an additional dimension to binding in which dynamism possibly plays a role in modulating proteome-wide interactions [89].

The two prevalent models of protein binding introduced above (Figure 1)—“lock and key” [33,34] and “induced fit” [35]—have worked remarkably well in explaining the behavior of well-folding proteins [2,3]. These traditional binding modes, however, can not account for proteins that display high structural dynamism, for which a new set of binding models have emerged (Figure 2c,d). These “newer” models that accommodate the behavior of hyper-dynamic proteins/regions will be discussed below.

### 3.1. Binding Model #3: Conformational Selection

Important to the lock-and-key model is the idea that particular conformations bind to particular partners. For structurally dynamic proteins, each of the conformations available in their structural ensembles, may potentially bind to a specific binding partner. In this dynamic binding model—now known as “conformational selection” (Figure 2c; reviewed in [10])—a binding partner would, during binding, stabilize a particular (presumably complementary) and already preexisting conformation within the protein’s ensemble, thereby shifting the conformational population within the ensemble. Examples of conformational selection are found in the binding of ubiquitin [52,53] and immature (germ line) antibodies [82,90], which appear to display multiple unbound conformations that correspond to complements of specific binding partners. These examples raise interesting connections between conformational diversity (or structural dynamism) and functional/binding *promiscuity*, which is discussed soon.

*“Conformational selection” is the same as “fluctuation fit” from the 1960’s.* It is noteworthy that this dynamic mode of protein binding is not as new as it might seem, as elements of this model (e.g., ensemble degeneracy [11,12] and conformational selection [12]) were brought up by Landsteiner [11] and Pauling [12] in their 1930’s trailblazing attempts at understanding the binding versatility of antibodies. Additionally, shortly after “induced fit” contended for the textbooks [35], Straub and colleague synthesized a prototype of today’s conformational selection, which he then called the fluctuation fit model [91,92]. The mechanism of fluctuation fit, unfortunately, could not be experimentally resolved from that of induced fit at the time of its introduction [10], and so these concepts were left dormant until further and more discerning experimental and computational techniques emerged [10]. From Straub till today, this dynamic mode of binding has gathered many monikers some of which are: “fluctuation fit”, “conformational selection”, “conformational selectivity”, “population shift”, “selected fit”, “stabilization of conformational ensembles”, and “preexisting equilibrium” (reviewed in [10]). However, given that Straub’s “fluctuation fit” accommodated only “nearly identical” conformations within the ensemble [92], this review sticks with what appears to be the popular term of today: “conformational selection.”

### 3.2. Promiscuous Binding

Early accounts arising between the 1930s [11,12,48,49,50] and 1960s [91,92] recognized that dynamic proteins that display more conformations within their ensembles pose the possibility of binding to multiple partners. This mode of thinking, however, took a back seat to single-conformation theories (such as lock and key and induced fit; both of which have one dominant conformation in their unbound states), particularly because more dynamic theories could not be experimentally validated till only relatively recently [10]. Today, a number of promiscuous binders have been found [9,13,19,20,93], and the link between dynamism and promiscuity is now firmly established [19,20,94] (of course, large proteins may also attain binding “promiscuity” by displaying multiple binding domains). This link provides a richer picture of how evolution may progress at the molecular level (discussed soon).

### 3.3. Binding Model #4: IDPs Often Bind by Losing Structural Diversity

Being on the extreme spectrum of structural dynamism (Section 2), IDPs have stretched the utility of all contemporary structure/binding models. While some IDPs and IDRs (intrinsically disordered regions) are shown to remain dynamic even when bound to their partners (discussed in Section 3.4), many IDPs bind their partners with a concomitant reduction in the conformational ensemble size. Two main models exist for this reduction or “collapse” in structural ensemble size: (1) by the previously discussed mode of “conformational selection”, where conformations present within the unbound ensemble partake in binding (Figure 2c); and (2) by a folding (or collapsing) event induced by a binder (Figure 2d). The difference between the two processes is that in the former mode, the binding conformation is already available within the pre-bound ensemble, and in the latter mode, the bound structure does not exist pre-binding, but is induced into forming.

*Conformational selection in IDPs.* Evidence for conformational selection can be found in a number of IDPs/IDRs [62]. For example, the C-terminus portion of Ubiquitin is an IDR that, even in the unbound state, cycles through conformations that appear similar to those when bound to specific partners [52,53].

*Induced folding or the fly-casting model in IDPs.* “Induced folding” is evidenced in cell cycle inhibitors such as p27(Kip1) [83,84,85] and the transactivation domain of p53 [86]. In this mode of binding—sometimes likened to “fly-casting” [95,96]—the binding partner comes into contact with small parts of the mostly disordered (but residually and locally structured [83,85,97,98]) protein, followed by the evolution of a stronger binding pose accompanied by a folding event (Figure 2d) [62,96]. At least three features are expected to be true for this mode of binding: first, the local residual structure such as helices [83,85,97] potentially help in binding events; second, on account of being extended, the effective capture radius of the protein is increased [95]; and third, the entropic cost of binding is potentially defrayed by the coupling of binding to IDP folding [99]. While large capture radii would increase binding rates, an important counter to this effect—an IDP’s potentially small translational diffusion constants—might counter the benefits of the larger capture radius [99]. The interplay of all these properties (effective capture radius, diffusion constants) and events (binding, folding) is potentially complicated (and possibly depends on the specific protein), and so the question of which of the properties allows for IDPs to efficiently bind to their targets is still being explored.

### 3.4. Binding Modes Describe a Spectrum, as Do Bound Complexes

Just as protein dynamism may be described as a continuous spectrum of states, and not just as “rigid” and “flexible”, the modes of protein binding, facilitated by the state of the pre-bound protein’s dynamism, could function using a spectrum or combination of binding modes [100]. For example, even a lock-and-key binder is expected to accommodate *some* change in the states of sidechains at the binding site (which is a feature of induced fit). Similarly, even conformational selection (the dynamic analog of lock-and-key) is expected to conform to an extent to a bound partner [101] (which is the hallmark of induced folding [62,96] and induced shift [81,102] modes of binding). To provide a concrete example, the measles nucleoprotein first undergoes a minor conformational selection with its binding partner (via transient helical conformations) after which an induced folding event occurs [103] (in a manner that is similar to but not the same as fly-casting [95] discussed in Section 3.3). In one sense, the pedagogical models of binding should be considered to be more like instructional lessons/caricatures or rules of thumb; since the lines distinguishing each binding mode will inevitably be blurred by unruly, messy, real proteins.

*Fuzzy complexes* (Figure 2(*iii*))*.* So far, participants in the binding models described above (Figure 2a–d) presumably display a single dominant conformation per bound complex. However, Tompa and Fuxreiter showed that a number of binding partners interact in more than one conformation [89] and sometimes even are disordered in the bound state [71,88,89,104,105]. Like the spectrum of structural states and binding modes (discussed above), disorder in bound states—“fuzziness” [88,89]—also takes on a spectrum of possibilities from those complexes displaying little disorder (e.g., in lock and key binders), segmental disorder (e.g., linkers in bipartite clamps [105]), and even complete disorder or high “fuzzyness” (e.g., some histone acetyl transferase-associated proteins [87]). This description is related but not identical to binding modes, and was proposed [89] to help characterize the interactome (the network of protein-protein interactions within an organism).

### 3.5. Sequence Determinants (and Bioinformatics) of Promiscuous Function and Dynamic Structure

Before drawing connections between structural/functional promiscuity and evolvability (Section 4), this section steps back and visits the question of how sequence determines a protein’s binding promiscuity and dynamic nature.

*Binding promiscuity determinants.* Anecdotal studies show that binding promiscuity obtained by structural disorder may occur on the two extremes of the hyrophobicity scale: (1) on the low hydrophobicity end (and concomitantly, high polar and charged end), solvated and relatively extended segments of proteins are likely to display multiple conformations useful in binding (e.g., [9,106]); and (2) on the high hydrophobicity end, hydrophobic interactions are known to be non-directional or degenerate, which is a common prerequisite to promiscuity (e.g., patches of hydrophobic residues are implicated in promiscuous protein-protein interactions [93] and enzymatic reactions [19,94,106]).

*Intrinsic disorder determinants.* It is interesting that the former *general* sequence determinant of binding promiscuity (low hydrophobicity and, consequently, high polar/charged groups) also are characteristics of sequences describing intrinsic disorder [21]. Particularly, Uversky *et al*. [14,64] used sequence analysis to show that IDPs were distinguished from folding proteins by displaying both low hydrophobicity and high net charge *magnitude*, both of which would presumably prevent hydrophobic collapse [107]. Additionally, IDPs may be separated into at least two structurally distinct classes—those that describe “random” coil (or extended) states with high stokes radii, and those that describe more compact “premolten” globules that display stokes radii between the canonical molten globule and purely extended states [14]. Interestingly, limited but intuitive observations [108] indicate that these two classes of intrinsic disorder may be distinguished by the percentage of charged amino acids [%ERDK] in the sequence divided by the percentage of hydrophobic amino acids [%FILVWA] within the sequence [108], with higher values describing extended/relaxed coils and a threshold ratio (approximately between 0.5–0.6) triggering the transition from premolten globule to coil [108].

While important in establishing sequence determinants for intrinsic disorder, these bioinformatics programs [4,15,18,63,64,65,66] have truly revolutionized our understanding of just how prevalent IDPs and IDRs are in proteomes and life processes. For example, more than ∼25% or more of eukaryotic proteins are mostly disordered [4,18], and more than 50% of eukaryotic proteins possess IDRs. These findings have helped propel *un*structural biology from the fringes to an increasingly brightening spotlight [15,22].

## 4. Third “New View”: The Role of Protein Dynamism in Evolution

As discussed above, structural dynamism has greatly expanded our picture of what proteins “look like” (Section 2) and how they often function promiscuously (Section 3). Given the intimate link between function and evolution it follows that protein dynamism finally must affect protein evolution.

### 4.1. Dynamism and Promiscuity Hastens Evolution of New Functionality

The idea that structural/functional promiscuity [94] might allow for higher evolvability has existed in some form since the 1930’s, when Landsteiner [11] and Pauling [12] (reviewed in Ref. [16]) puzzled over how antibodies could eventually bind strongly to virtually any molecular partner (hapten). The results of their work will be discussed shortly as an illustration of all salient points of this review (Section 5). Regarding the relationship between promiscuity and evolvability, however, their work presaged what is now becoming more and more apparent: dynamism provides a protein with conformations alternative to the functional conformation, and each conformation represents a potential new function [13,16,94] for adaptive evolution to work on. Especially interesting is the idea that new functionality (and configurations) can accumulate with little loss of original functionality, e.g., in the evolution of hormone receptors [109,110] and directed evolution of transcription factor effectors [111].

How does this conformational diversity come to exist? How specifically does it affect evolution? These questions ultimately must dovetail with the nature of sequence evolution, as the evolution of protein sequence, structure and function must be thought of as facets of the same evolutionary phenomenon. For that reason, the history and present understanding of sequence evolution must be assessed before making final connections.

### 4.2. Molecular (Sequence) Evolution from a Historical Perspective

In the early 1960’s, the first wave of sequenced proteins provided the first important rule of molecular evolution: *sequences of functionally similar proteins can be highly dissimilar or divergent* [112]. While today this notion is the norm (and is the basis for molecular clocks [112,113,114,115] and phylogenetics [116]), then, however, the prevalent view expected that the rate of evolution (molecular or otherwise) is dominantly dependent on natural selection, *i.e.*, proteins with conserved folds and functions should have equally conserved sequences (discussed in Refs. [117,112]). Contrary to this view, while protein structure and function was found to negligibly change, their sequences were evolving at a rate that you could set a proverbial evolutionary clock to (something that came to be called the molecular clock hypothesis [113,114,115]).

**Figure 3:**
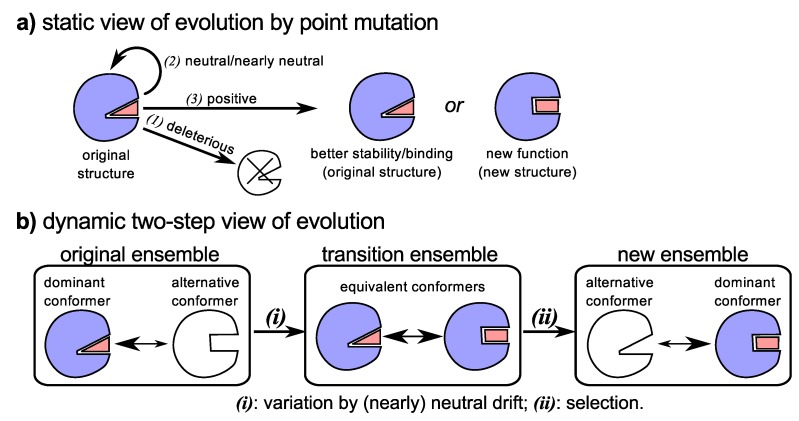
The traditional/biochemical view (**a**) *versus* the new view (**b**) of the evolution of new functionality and binding partners. Traditionally (**a**), mutations that facilitate sequence evolution allowed for three outcomes: (*1*) death due to a deleterious mutation; (*2*) no change in fitness or protein functionality/stability due to a (nearly) neutral mutation; and (*3*) increase in organismal fitness due increase in the stability of the original structure, improvement of original function, emergence of new functionality, *etc*. [2]. Today, dynamism and neutral drift provide an integrated picture of how new functionality might appear (**b**): (*i*) neutral drift maintains the proteins original functionality while either maintaining or increasing dynamism that supports promiscuous functionality; and (*ii*) with the help of gene duplication, alternative and novel functionality would be selected due to happenstance pressures.

To explain the relatively rampant rate of sequence evolution, neutral [118,119,120] (and nearly neutral [121,122,123,124]) evolution was introduced. In these models, sequences dominantly evolve by either neutral [118,119,120] or nearly neutral [121,122,123,124] (possibly slightly deleterious) mutations that do not substantially affect the protein’s structure and function [125], and are tolerable due to the evolving organism’s population size. Of course, (nearly) neutral evolution can not solely exist, as adaptive mutations allowing for gain of function had to have occurred at some time [126,127,128,129]. Despite the strong (and sometimes loud [130]) debates regarding the relative importance of neutral *versus* adaptive mutations, the resulting picture that emerged over the decades is qualitatively the same (Figure 3a): (1) neutral and nearly neutral evolution provides a steady accumulation of mutations that do not perceptively modify protein structure and function [118,119,120,121,122,123,124]; and (2) episodic mutations associated with adaptation occur during events such as speciation due to environmental selection factors [126,127,128] that possibly substantially modify perceived mutation rates [131,132,133,134,135,136], protein stability, structure and function.

The view discussed above, that neutral mutations occur at a steady state (like a ticking clock), while adaptive mutations come and go episodically, has been a cornerstone of sequence evolution. However, the re-discovery of the role of structural dynamism and functional promiscuity in evolution (discussed above), along with the role of neutral sequence evolution in facilitating states of dynamism and promiscuity, has allowed for some illuminating links between two seemingly disparate and hotly debated viewpoints of neutralism and non-neutralism.

### 4.3. Neutral Drift Increases Evolutionary Fodder (Structural Dynamism and Functional Promiscuity)

While neutral and adaptive sequence evolution may explain some observations, an outstanding question, among others [124], remains: does neutral drift play a part in the evolution of structure/function? The recent progress and new views of structural and functional promiscuity have allowed for this question to be more readily accessed. Particularly, it is being found that while neutral evolution may not change the primary function (and dominant conformation) of a protein [25,137,138], it does appear to help enrich the presence of alternative conformations in the protein’s structural ensemble [25,137,138,139], which, as we have seen in Section 4.1, serves as a nursery for the emergence of *potential* alternative functionality. The importance of this statement requires reiteration: The accumulation of neutral mutations—neutral drift—allows for the generation of genetic diversity that provides a rich structural reservoir for the evolution by adaptation of novel molecular functionality [25,138], while maintaining the structural requirements of the protein’s primary function [25,138].

Finally, the historically warring [130] sects of sequence evolution—“neutral” and “adaptive” evolution—are married together as two crucial and inalienable components of the same process (reviewed in [25]): (1) the accumulation of neutral or nearly neutral mutations allowing for greater structural variability (Figure 3b-(*i*)); and (2) the happenstance recruitment of an alternative conformation to perform an alternative function (Figure 3b-(*ii*)). Combining both neutral *and* adaptive mutations in the evolution of novel functionality (along with with processes like gene duplication), the troubling “chicken-egg” puzzle of how structure must match an unmet function is quelled, since the structure was already present in residual amount.

## 5. Antibody Maturation: A Single System Describing Many Crucial Elements of Dynamism

Antibodies deserve an entire section for two reasons: (1) the historical work on antibodies in the 1930’s [140] presaged many of the new discoveries made on dynamism this and last decade; and (2) while most other examples of evolution are organismal, antibodies undergo accelerated evolution within a single organism (via somatic hypermutation [141,142]), thereby allowing us to witness the “natural” evolution of a specific binding function.

### 5.1. Possibly the First Reference to IDPs and IDRs

Even in the 1930’s, it was evident that a repertoire of limited antibodies could bind to almost *any* foreign molecule (antigens/haptens) [11,12,48,49,50]. How could such diversity be possible with protein sequences that were obviously limited in combinations and extent? Two notable models were proposed based on the capacity of one sequence to display multiple conformations; and while only one model stood the test of scrutiny (historically discussed in Ref. [140], pp. 127–131), both are remarkable in anticipating the two types of proteins that would not be truly recognized until the turn of the century. While both models were discussed in distinct terms, they will be referred to using today’s terms for the sake of consistency.

The first antibody-antigen binding model was proposed by Breinl [48], Alexander [49] and Mudd [50] between 1930 and 1932. This model generally assumed that antibodies are intrinsically disordered proteins (discussed in Section 2) that promiscuously bind to their myriad partners (antigens) via an induced folding event (Section 3). Recognizing the omission in the first model—that only part (not all) of the structure appeared “hesitant” or disordered–Landsteiner [11] and Pauling [12] proposed that antibodies displayed intrinsically disordered *regions* (now called hypervariable regions) that displayed numerous conformations within their structural ensembles, each with the possibility of binding distinct partners (antigens). It is fascinating that these models anticipated almost all the “alternative” models of structure and function decades before textbook models were even established.

### 5.2. Antibody Structure and Function Today

More than seven decades later, Landsteiner and Pauling’s model of antibodies [11,12] serves as an exemplar of the new views discussed here. For example: (**1**) the hypervariable region (HVR) of the antibody is essentially intrinsically disordered [143,144], as predicted [11,12]; (**2**) HVRs of immature antibodies attain binding promiscuity through *conformational selection*, as distinct conformations within the ensemble bind distinct partners [82,90]; and (**3**) during an immune response, immature antibodies proceed from being promiscuous to specific binders in a process called affinity maturation. In one instance, affinity maturation has been shown to occur by reducing the structural dynamism of the HVR through sequence mutations [143,144]. This is an excellent example of how, by tuning the dynamism of the protein (Figure 2(*i*)), a binding mode is tuned also (Figure 2(*ii*)).

## 6. From Protein Evolution to Protein Origination

### 6.1. Differentiation

**Figure 4:**
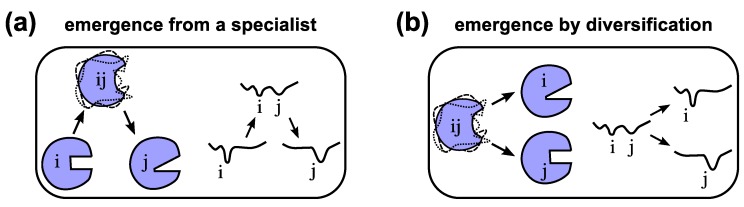
Models of the evolution of new functionality. Emergence of a protein displaying new functionality “j” is possible either by emerging from an existing “specialist” protein “i” [20] through a dynamic and promiscuous/ambiguous intermediate “ij” (**a**), or by emerging through differentiation [145] or specialization from a protein of promiscuous functionality “ij” (**b**) also see Figure 3 in Ref. [20]. Arbitrary conformational free energy landscapes shown.

The evolution of one specialized functional protein from another through a promiscuous intermediate (Figure 4a) indicates a “serialness” to the process that may not exist in all situations. A particularly interesting hypothesis proposed by Jensen in 1976 [145] turns this scenario on its side (Figure 4b): assuming that ancient organisms have smaller genomes and protein repertoires [146], these proteins with less specialized and potentially ambiguous functionality could diversify into a number of specialized enzymes, with the help of gene duplication and sequence evolution [13,145]. This mode of evolution also called the “differential narrowing of substrate specificities” [147] or differentiation, is expected to lead to proteins of diverse families and superfamilies today [147,148,149]. Both the serial (Figure 4a) and divergent (Figure 4b) modes of protein evolution are models of protein evolution that require the existence of a fully functioning parent protein [145,147]. At one point in evolution, however, proteins would have had to originate from random peptides.

### 6.2. Origination

How did the first protein folds come to exist? This question is especially interesting to the origins of life, given that (1) protein folds are extensively utilized by simpler organisms (e.g., bacteria) for sustaining life; and (2) random peptides are expected to have existed well before the appearance of evolved organisms (given the abiotic presence of amino acids [150,151,152,153,154] and peptide bond formers [155,156,157]).

**Figure 5:**
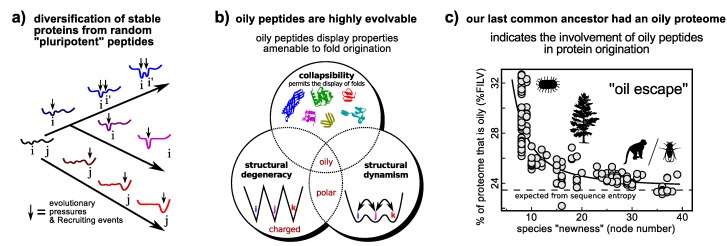
A model of protein origination that employs dynamism. The differentiation model that utilizes a promiscuous and presumably dynamic protein as an ancestor (Figure 4b) may be extended to the origination of a functional folding protein from a random “pluripotent” peptide (**a**). Utilizing the link between promiscuity and evolvability (Section 4.1), certain classes of random peptides, such as oily peptides (those that display high %FILV [160]), display properties that would allow for the enrichment of folded conformations within its ensemble (**b**) [160], thereby making them potentially superior substrates for *ab initio* protein fold invention [160]. Interestingly, this prediction is supported by the finding that our last common ancestor’s proteome had an oily beginning, as evidenced in the strong negative correlation between a species’ proteome oil content and the extent (node number) to which it is expected to have diverged from the last common ancestor (**e**). The hypothesis highlights the utility of dynamism (this time extreme dynamism) that could help in formulating simple tests of how proteins came to be. Panels **a**/**b** and panel **c** were adapted from Refs. [160,161] (reviewed in [159]), respectively. Arbitrary conformational free energy landscapes shown in panel **a**.

The “new views” of protein dynamism allow us to cast a fresh perspective on the question of protein fold origination. Particularly interesting from the discussions on dynamism/promiscuity/evolvability is the idea that while the probability of encountering a random peptide sequence that folds well is vanishing [158], the probability of encountering random peptides of particular properties (Figure 5b) that *transiently* display novel folded structure and functionalities is much higher. This idea is an extreme version of Jensen’s differentiation model (Figure 4b) [145], and is called the “pluripotent hypothesis” on account of the random peptide’s potential to be evolved into one of many transient folds [159].

The interesting part of the hypothesis is that oily peptides (those described as having high %FILV [160]) indeed possess all of the properties (e.g., foldedness and dynamism; Figure 5b) important for protein fold origination [160], thereby allowing for simulation [160] and bioinformatics [161] to test the hypothesis. The following recent findings are in agreement to the pluripotent hypothesis: (1) oily peptides are, indeed, superior substrates for directed evolution to “evolvable” folds [159]; and (2) bioinformatics on whole proteomes [161] indicates that the last common ancestor’s proteomes had higher than average oil content (Figure 5c).

This hypothesis is one among many possible routes to protein invention; however it is so far the only one backed by fossil/trace evidence in proteomes [161] (Figure 5c). Another mode of evolution—one that depends on the origination of all proteins from early intrinsically disordered proteins—remains a tantalizing possibility. So far, however, the following evidence works against this route as a contender: (**1**) extended proteins in unprotected and chemically reactive environments are exposed to chemical degradation [158]; (**2**) extended proteins—IDPs and IDRs—rise in prominence only later on in the evolution of complex organisms[4]; (**3**) compositions accommodating IDPs and IDRs (high charge and polar content) are also inefficient at directed evolution into folded proteins of good design (something that oily peptides excel at even more than other well-folding proteins) [160]. No theory, however, must be discounted at so early a stage in our investigation of how functional proteins emerged.

## 7. New Connections and Questions: Links to the Advent of Complex Organisms and Diseases

As reviewed above, the introduction of disorder to the field of protein science has permitted many molds to be broken on how proteins traditionally look, function and evolve. While well-folding proteins perform the role of the fastidious and relatively specific catalyzers (or binders), IDPs and IDRs perform the role of the generalist that each display a broad spectrum of conformations and binding capabilities [162]. This binding promiscuity, while relatively unimportant when thinking of a protein in isolation, becomes a crucial aspect when considering the cell as a collection of biomolecular interactions.

*Promiscuity and complex life.* Particularly, the increase in intrinsic disorder within proteomes is linked to the increase in organismal complexity (e.g., ∼5% of bacterial proteins are predicted to be mostly disordered, while ∼25% or more of eukaryotic proteins are mostly disordered [4,18]), possibly because of multifarious roles afforded by promiscuity that would allow for modularization of protein interaction networks [163,164,165]. This association is strengthened with the observations that (**1**) IDPs and IDRs are associated with a number of cell signaling activities; (**2**) the number of “hub proteins”—those described as highly promiscuous binders in an organism—is positively correlated with organismal complexity; and (**3**) hub proteins are often characterized as containing intrinsically disordered regions [163,164,165]. The association of IDPs/IDRs with complex life is made stronger with the connections between IDPs/IDRs and diseases associated with complex organisms such as cancer [6] and Parkinson’s disease [7]; yet little is known about how intrinsic disorder came to be utilized by complex life [4].

## 8. Final Words

The tantalizing connections between disorder and both complex life and disease assure sustained future investigations into the role of structural disorder in proteins. As new ideas subsume old ones in the field of structural and *un*structural biology, I will end with a French phrase—originally used for the accession of a new monarch—that indicates continuity and change at the same time: “*la structure est mort, vive la structure!*” (the structure is dead, long live the structure!)
